# Anxiety disorders in headache patients in a specialised clinic: prevalence and symptoms in comparison to patients in a general neurological clinic

**DOI:** 10.1007/s10194-011-0293-9

**Published:** 2011-02-06

**Authors:** D. Mehlsteibl, C. Schankin, P. Hering, P. Sostak, A. Straube

**Affiliations:** Department of Neurology, Ludwig-Maximilians-University Campus Großhadern, Marchioninistraße 15, 81377 München, Germany

**Keywords:** Headache, Generalised anxiety disorder, Panic disorder, Neurological patients, Drug use

## Abstract

Data from several studies indicate an association of headache with anxiety disorders. In this study, we assessed and differentiated anxiety disorders in 100 headache patients by using the PSWQ (Penn State Worry Questionnaire) screening tool for generalised anxiety disorder (GAD) and the ACQ (Agoraphobic Cognitions Questionnaire) and BSQ (Body Sensation Questionnaire) for panic disorder (PD). Control groups were constructed: (1) on the basis of epidemiological studies on PD and GAD in the general population and (2) by including neurological patients. 37.0% of headache patients had a GAD. 27% of headache patients met the score for PD in the BSQ, 4.0% in the ACQ. Significant results were obtained in comparison to the general population (*p* < 0.001) and with regard to GAD in comparison with a sample of neurological patients (*p* < 0.005). The BSQ significantly correlated with the number of medication days (*p* < 0.005). The results confirm the increased prevalence of GAD in headache patients. PD seems to increase the risk of medication overuse.

## Introduction

The increased comorbidity of mental disorders and headache has been demonstrated by several epidemiological studies [1, 2]. These studies often focus on the association of depression and headaches. However, there are indications that the risk of suffering from an anxiety disorder with coexisting headache is higher than the risk of suffering from a depression [3, 4].

Clinically, it is important to make a distinction between the different anxiety syndromes. Migraine patients’ risk of suffering from a panic disorder (PD) is up to ten times higher than that of the general population [5]. Patients with migraine and chronic headache have a four to fivefold increased risk of suffering from generalised anxiety disorder (GAD) [5, 6]. Some studies have also found that headache patients suffer more frequently from GAD than from PD [7]. Given the current state of research, the findings are heterogeneous. In addition, only a few studies specifically examine what specific type of anxiety disorder headache that patients suffer from [8]. This is due to the fact that screening for anxiety is more difficult than screening for depressive disorders because anxiety has many manifestations and the physical symptoms of anxiety are similar to symptoms of somatic diseases. A disorder-specific diagnosis is useful because different anxiety disorders have a different phenomenology and thus require differential (pharmacological and psychotherapeutic) treatment.

The possible presence of a co-morbid anxiety disorder is important for various reasons. First, the prognosis is more unfavourable when an anxiety disorder occurs simultaneously with headache [9]. Second, there are significantly higher costs for the health system if there is a co-morbid anxiety disorder [10]. Third, quality of life is significantly more impaired when an anxiety disorder and headache are co-morbid than when only one disease is present [11]. The probability of a simple migraine developing into a transformed migraine is increased if there is a co-morbid mental disease [12]. Headache patients appear to suffer from a co-morbid mental disease more frequently than general neurological patients [13, 14]. Little is known about the specific anxiety disorders in headache patients.

This prospective study aims to investigate the prevalence of PD and GAD among a group of patients in a tertiary headache clinic. The results are compared to the prevalence of these anxiety disorders in the general population and in outpatients not suffering from headache. We further explore whether there is a relationship between these anxiety disorders and the amount of pain medication used by headache patients.

## Methods

### Study participants

We prospectively recruited patients presenting between April 2009 and October 2009 at our outpatient tertiary headache centre. A randomized age- and gender-matched control group (*n* = 20) was drawn from the non-headache outpatient clinic of our neurological department. The exclusion criterion for both groups was the existence of a depressive disorder (ICD-10: F32–F38 diagnoses), with the additional exclusion criterion of any history of headache and dementia for the control group. All patients gave their informed consent prior to their inclusion in the study.

### Characteristics of headache patients

The majority of the 100 headache patients included in the study was female (37.2 + 12.4 years). 8% of patients were male (44.0 + 15.9 years). 70% (*n* = 70) of patients suffered from episodic migraine without aura (MoA), 10% (*n* = 10) had episodic migraine with aura (MA), 3% (*n* = 3) had tension-type headache (TTH) and medication overuse headache (MOH) was present in 17% (*n* = 17) of patients. 39.0% (*n* = 39) of patients with migraine (MoA and MA) fulfilled the criteria for chronic migraine according to the appendix criteria of the International Headache Society (IHS) 2006 [15].

### Characteristics of neurological patients

80.0% of the 20 patients in our control group were female (41.6 + 11.4 years), 20.0% were male (40.4 + 13.0 years). Table 1 shows the neurological diseases of the control group.

**Table 1:** Diagnoses of the control group from the outpatient clinic at the department of neurology

Diagnosis (ICD-10)	*n* (%)
Myopathy G72.9	1 (5.0%)
Intracerebral haemorrhage in hemisphere, unspecified I61.2	1 (5.0%)
Encephalitis, myelitis and encephalomyelitis, unspecified G04.9	2 (10.0%)
Paraesthesia of skin R20.2	1 (5.0%)
Cerebral infarction due to unspecified occlusion or stenosis of cerebral arteries I63.5	1 (5.0%)
Cervicobrachial syndrome M53.1	1 (5.0%)
Spinal stenosis M48.0	1 (5.0%)
Other and unspecified abnormalities of gait and mobility R26.8	1 (5.0%)
Spastic diplegic cerebral palsy G80.1	2 (10.0%)
Vascular myelopathies G95.1	1 (5.0%)
Aneurysm of carotid artery I72.0	1 (5.0%)
Meningitis in bacterial diseases classified elsewhere G01	3 (15.0%)
Carpal tunnel syndrome G56.0	1 (5.0%)
Other specified endocrine disorders E34.8	1 (5.0%)
Hereditary ataxia, unspecified G11.9	1 (5.0%)
Neuroborreliosis G69.2	1 (5.0%)

*ICD* International Classification of Diseases

## Material

The following standardized disorder-specific self-report instruments were used:

(1) Screening for GAD—Penn State Worry Questionnaire (PSWQ) [16]

This questionnaire quantifies pathological worry which, according to DSM-IV, constitutes the cardinal feature of GAD. It is a 16-item self-report measure of worry. The items of the PSWQ are rated on a five-point Likert scale. The cut-off value is a score of 49 in women and a score of 45 in men. Pathological worry is defined as a one-dimensional concept. The measure has been shown to have excellent psychometric properties in clinical and non-clinical samples; the internal consistency (Cronbach’s alpha = 0.86–0.95) is good [17, 18].

(2) Screening for PD—Body Sensations Questionnaire/Agoraphobic Cognitions Questionnaire [19]

The BSQ contains 17 items concerning the degree to which patients fear somatic symptoms commonly associated with anxiety and panic attacks. The inventory assesses the fear of certain body sensations. Items are rated on a five-point Likert scale ranging from 1 = “not frightened or worried by this sensation” to 5 = “extremely frightened by this sensation”.

The ACQ measures the frequency of fear-related cognitions, the fear of negative social or health consequences of fear. The ACQ contains 14 items (6 behavioural-social and 8 physical items) rated on a five-point Likert format, ranging from 1 = “the thought never occurs” to 5 = “the thought always occurs when I am nervous”.

The mean of all items was calculated for the evaluation. The cut-off is 2.3 for ACQ and 2.5 for BSQ. The internal consistency (Cronbach’s alpha = 0.80–0.95) for the BSQ is good; the internal consistency for the ACQ (Cronbach’s alpha = 0.74–0.87) is satisfactory. The retest reliability is between *r*_tt_ = 0.63 and 0.8.

On the basis of current national epidemiological studies on PD [20] and GAD [21], the expected prevalence of each anxiety disorder was calculated for an age- and gender-matched group of the general population. Both studies presented their results in four age and gender groups. The number *n* of headache patients was identified for each class. Then the prevalence *P* of each anxiety disorder was obtained for each sex and age group in the literature. Finally, the expected number of patients with GAD and PD was calculated for the general population by multiplying *n* by *P*.

The estimation of dosages of acute medication of the patients was done based on the detailed medical charts and personal examinations and interviews.

This study was conducted in accordance with the ethical principles of the Declaration of Helsinki.

### Statistical analysis

Statistical analysis was performed using SPSS 17.0 for Windows. Descriptive statistics consisted of frequency counts and percentages or means and standard deviations. Binary or ordinal factors were analysed with Chi-square for comparisons between groups. To establish the relationship between anxiety disorders and medication use behaviour, the Spearman correlation coefficient was calculated. Significance was set at *p* < 0.05 in all cases.

## Results

### Anxiety disorders

**Fig. 1:**
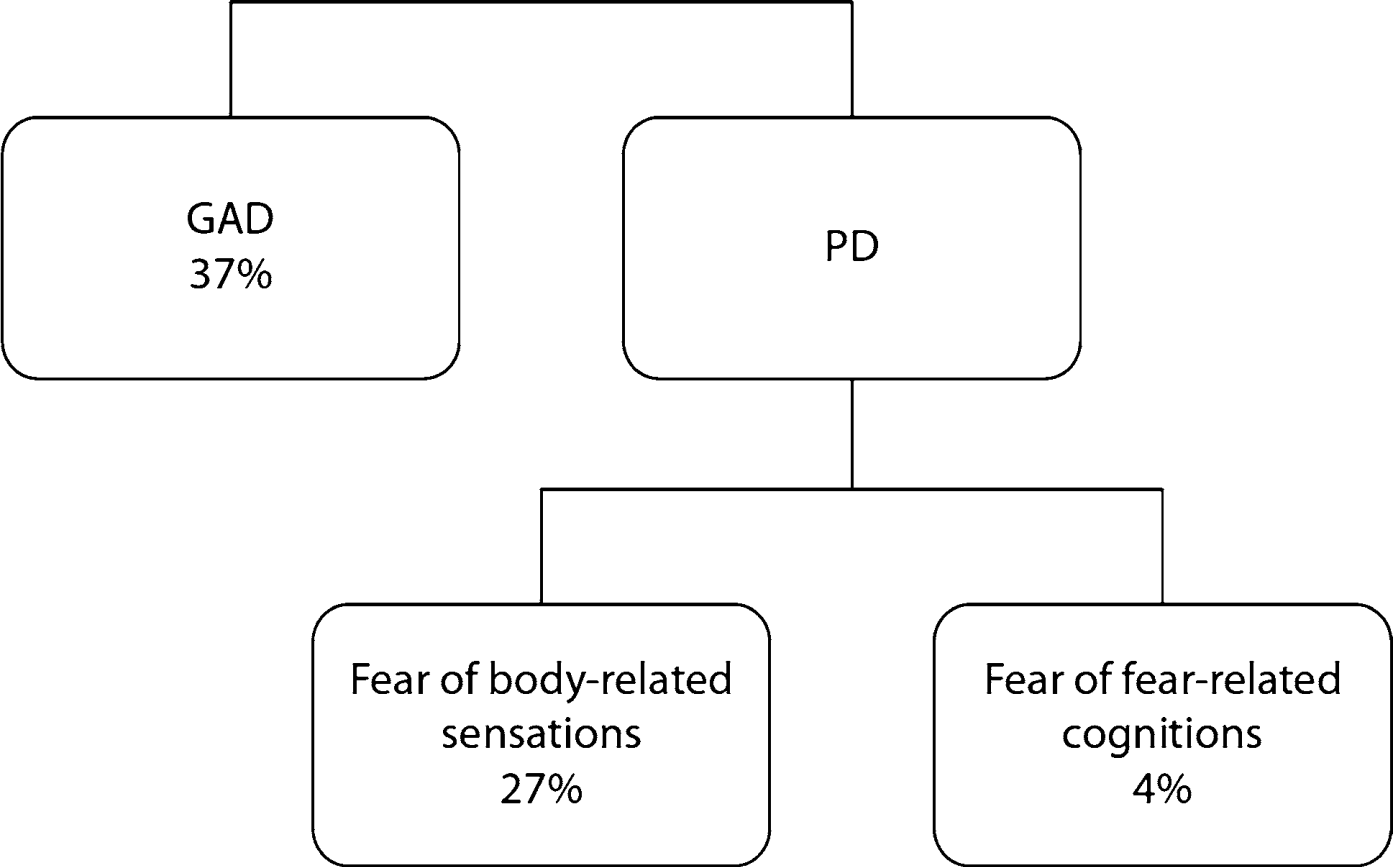
Distribution of specific anxiety disorders in the population of headache patients

37% (*n* = 37) of the headache patients (*n* = 100) also suffered from GAD as measured by PSWQ, 27% of headache patients had a BSQ score above ≥2.3 indicating a PD concentrating on body-related sensations, while 4% had a fear of fear-related cognitions, as measured by the ACQ (see Fig. 1). A striking result of the screening is that the combination of both anxiety disorders was found in 13% of the headache population.

**Fig. 2:**
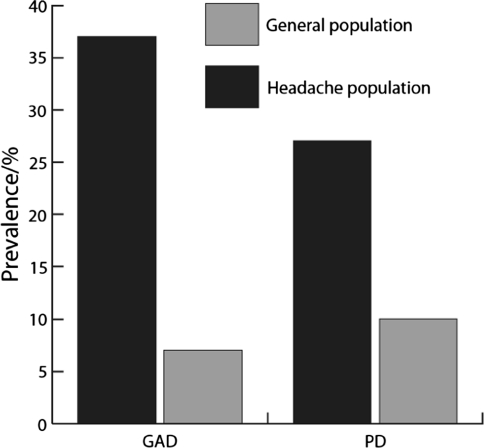
The prevalence of GAD and PD differs significantly (*p* < 0.01) in patients with headache compared to the general population

The group of headache patients (*n* = 100) differed significantly from the estimated general population (*n* = 100) in regard to the manifestations of the anxiety disorders: this means (χ^2^ = 26.22;* df* = 1; *p* = 0.000) for GAD and (χ^2^ = 9.58;* df* = 1; *p* = 0.002) for PD (see Fig. 2).

**Fig. 3:**
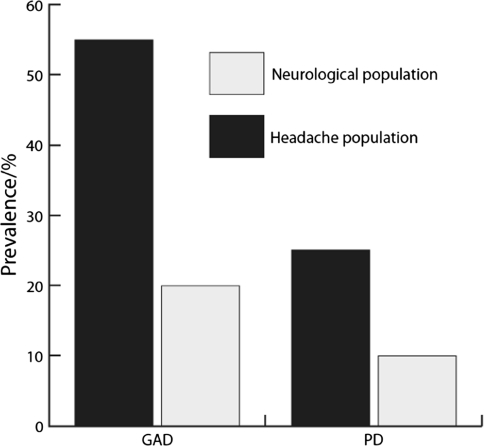
The prevalence of GAD differs significantly (*p* < 0.05) between headache patients and patients with non-headache neurological diseases. The prevalence of PD does not differ significantly between the two groups

The proportion of patients suffering from GAD was significantly higher among headache patients (*n* = 20) than among neurological patients (*n* = 20) (χ^2^ = 5.23,* df* = 1, *p* = 0.022). The screening of these two populations for PD did not yield significant results (χ^2^ = 1.56,* df* = 1, *p* = 0.212) (see Fig. 3).

Headache patients took medication (triptan or analgesics) on 8.2 days per month on average. The correlation between the BSQ score and the number of medication days (*r* = 0.223, *p* = 0.028) was significant. In contrast, the correlation between the PSWQ (*r* = 0.90, *p* = 0.375) and ACQ scores (*r* = 0.64, *p* = 0.530) (*r* = 0.64, *p* = 0.530) and the number of medication days was not significant.

## Discussion

### Anxiety symptoms in headache patients

Despite the undisputed association between anxiety and headaches, many studies focus on the co-morbidity of depression and headache [22]. The lack of research on headache and anxiety disorder and the risk factors of this co-morbidity, such as chronification, increased risk of medication overuse, strongly reduced quality of life when headache and anxiety disorders coexist, make a differentiated investigation of anxiety disorders necessary. In addition, different treatment approaches are indicated, depending on what type of anxiety disorder is present.

We found that headache patients are more likely to suffer from GAD (37.0%) than from PD (27.0%). This result is confirmed by the HADAS study and a study by Merikangas et al. [4, 23]. Corchs et al. [24] found that 44.6% of chronic migraine patients also suffered from a GAD, corroborating the frequency of co-morbidity which we found in our study. Other studies demonstrated that PD is the most common anxiety disorder in headache patients [25]. However, different theoretical approaches indicate that GAD might be the more common anxiety disorder in headache patients. Recent theoretical conceptualizations of GAD suggest that “worry” (which constitutes the main feature of GAD) may be used to dampen emotional and somatic arousal [26, 27]. It is possible that headache patients may use worry as a strategy to reduce the somatic arousal that is associated with pain and as a result may become prone to developing GAD [22].

Furthermore, pathological worry as a main feature of GAD is similar to the cognitive style “catastrophizing”. The frequency of thought patterns with a tendency to catastrophize (e.g. anticipation of pain, rumination in response to the pain) has been shown for migraine patients [28] and other chronic pain syndromes (e.g. low back pain, rheumatoid arthritis) [29]. Catastrophizing is associated with a decreased level of functioning, i.e. more pain, more limitation by the pain and by pain behaviour and an increased use of professional medical services. There are other significant factors associated with GAD, which explain why GAD is the anxiety disorder which is most commonly found in this study. These consist of the knowledge that this anxiety disorder is relatively new, this disease pattern is often not diagnosed correctly and GAD patients typically consult primary care but rarely consult professional psychiatric or psychotherapeutic services [18]. This might explain the frequent occurrence of GAD in a somatic service centre such as the headache clinic.

In regard to PD, more patients scored higher on the dimension of fear of body-related symptoms (27% higher values in the BSQ) than on the dimension of fear-related cognitions (4.0% reached cut-off in the ACQ). This finding might indicate that particularly threatening assessment of physical symptoms and the resulting fear of physical symptoms in headache patients are more significant than the fear of the cognitive negative social or health consequences of fear. The threat of physical sensations, which is measured by the BSQ, is an aspect of anxiety sensitivity as, for instance, measured on the anxiety sensitivity index (ASI) that was originally conceived as an etiological factor in PD [19]. Drahovzal et al. [30] demonstrated that high ASI scores predict the occurrence of headache, pain intensity and other physical symptoms associated with headache. This result is remarkable as it was obtained in a non-clinical sample, and yet anxiety sensitivity is a predictor of headache. What both anxiety disorders have in common is the fact that they are not phobic anxiety disorders.

### Comparison of headache patients with the general population with regard to anxiety symptoms

For headache patients, the probability of suffering from GAD is about five times higher and the probability of suffering from PD is about three times higher than for the general population. This finding corresponds with the results of other studies that also found an increased prevalence of anxiety disorders in headache patients [31, 32]. Senaratane et al. [33] found that two-third of anxiety disorder patients suffered from migraine, with twice as many patients reporting migraine onset prior to the anxiety disorder than vice versa.

### Comparison of headache patients with neurological patients with regard to anxiety symptoms

More than 50% of headache patients exhibit indications of GAD in contrast to only one-fifth of neurological patients. The prevalence of PD did not differ significantly between the two groups, even if PD was found more frequently in the subpopulation of headache patients than in the group of general neurological patients. Other authors maintain the importance of anxiety symptoms in neurological patients [34, 35], but still there is not much data available [36]. Ekstrand et al. [13] found that 39% of neurological patients displayed an underlying psychiatric disorder: depression and somatoform disorder were the most prevalent disorders. Those with a psychiatric disorder were less likely to have an underlying neurological process as the cause of their symptoms. The results of our study show that headache patients in particular have an increased risk of having a psychiatric disorder. There are indications that co-morbid anxiety disorder in neurological patients is less adequately diagnosed and treated than co-morbid depression [37]. Particularly, psychiatric disorders among neurological patients increase the risk of a subsequent high number of non-psychiatric hospital admissions. Somatoform disorders, for example, have more than five times the risk for anxiety and depression with almost four times the risk for high use of hospital appointments [38]. Accordingly, the diagnosis and the treatment of anxiety disorders is important in neurological patients, and even more so in headache patients.

### Anxiety and medication use

Higher scores in the BSQ correlate significantly with the amount of medication taken during acute pain, but there is no correlation with the ACQ and the PSWQ. This might be explained by the fact that threatening physical symptoms are perceived as less threatening and an anxiety reduction occurs when medication is taken early. PD patients typically carry fear-reducing items (e.g. sugar, medicines, blood pressure monitor, and water), which can be seen on a behavioural level as an avoidance strategy. Frequent medication use also represents an avoidance strategy on a behavioural level. For GAD, the avoidance strategy is worry that takes place on a cognitive level.

### Neurobiological connections between anxiety and headache

Stress, strain and anxiety play an important role as trigger factors of migraine or can contribute to the disorder becoming chronic. One possible mechanism might be a sympathetic activation [39] that is common in both GAD and PD. It has been shown that high levels of fear and the experience of stress increase the perception of pain [40] and also that repeated experience of pain increases anxiety [41]. The experience of anxiety and pain may thus be considered as a reciprocal interaction. High scores on anxiety questionnaires are able to predict the activation of cortical areas responsible for pain perception [42]. From an evolutionary perspective, fear and pain can be classified as related qualities of experience which—as they serve as warning and protection—fulfil a function that is essential for survival. Knowing that cortical hyperexcitability plays a pathogenic role in migraine [43], a connection to GAD via noradrenergic dysfunctions is plausible. The locus coeruleus-norepinephrine axis seems especially important for the neurobiology of GAD [44]. The pharmacotherapeutic influence of this axis in the context of migraine prophylaxis with beta-blockers can reduce the frequency of migraine attacks by decreasing neuronal excitability [45]. It can be expected that there are common physiological processes. In the group of antidepressants, an action profile is documented for the serotonin–norepinephrine reuptake inhibitor for migraine prophylaxis as well as for co-morbid anxiety disorders [46]. On a genetic level, there is evidence that migraine and anxiety are associated by way of the s allele of the 5HTTLPR polymorphism of the serotonin transporter gene [47]. The importance of the 5-HTTLPR polymorphism is also demonstrated for PD [48].

### Limitations

Due to the relatively low group size, this study can only provide indications. The results should be confirmed in studies with larger samples. A further limitation is that the headache and the control group are almost exclusively composed of females. This applies particularly to the comparison with the control group of neurological patients. Another limitation is that the headache patients took triptans and analgesics, whereas the neurological patients and the subjects from the general population did not. The diagnosis of other anxiety disorders, such as specific phobia or social phobia, could also be important. In addition, there may be a bias with regard to the degree of psychopathology as the data were collected in specialised clinics.

## Conclusion

Headache patients suffer from anxiety disorders more frequently than the general population. GAD and the threatening assessment of somatic symptoms in the context of PD are of particular relevance. These symptoms may be directly related to an increased use of anti-pain medication. Compared to neurological patients, headache patients are more likely to suffer from a co-morbid anxiety disorder. Psychiatric diagnostics with a focus on anxiety disorders should therefore be established among neurological patients. The aim should be to initiate an appropriate therapy for anxiety disorders as soon as the disorder is detected in order to minimise the risk of chronification of headache and to prevent increased use of health care resources.
